# Predictors of Mortality among COVID-19 Patients Admitted to Intensive Care Units: A Single-Center Study in Tehran, Iran

**DOI:** 10.34172/aim.2022.106

**Published:** 2022-10-01

**Authors:** Arash Amini, Atabak Najafi, Arezoo Ahmadi, Mojtaba Mojtahedzadeh, Elahe Karimpour-Razkenari, Hamidreza Sharifnia, Shahriar Shahsavar Mistani, Farin Kamangar

**Affiliations:** ^1^Department of Anesthesiology and Critical Care, School of Medicine, Tehran University of Medical Sciences, Tehran, Iran; ^2^Department of Clinical Pharmacy, School of Pharmacy, Tehran University of Medical Sciences, Tehran, Iran; ^3^Department of Biology, School of Computer, Mathematical, and Natural Sciences, Morgan State University, Baltimore, MD, USA

**Keywords:** COVID-19, ICU, Iran, Mortality

## Abstract

**Background:**

Iran was one of the first countries to become an epicenter of the coronavirus disease 2019 (COVID-19) epidemic. However, there is a dearth of data on the outcomes of COVID-19 and predictors of death in intensive care units (ICUs) in Iran. We collected extensive data from patients admitted to the ICUs of the one of the tertiary referral hospitals in Tehran, Iran, to investigate the predictors of ICU mortality.

**Methods:**

The study population included 290 COVID-19 patients who were consecutively admitted to the ICUs of the Sina hospital from May 5, 2021, to December 6, 2021, a period that included the peak of the epidemic of the delta (δ) variant. Demographic data, history of prior chronic diseases, laboratory data (including markers of inflammation), radiologic data, and medication data were collected.

**Results:**

Of the 290 patients admitted to the ICUs, 187 (64.5%) died and 103 (35.5%) survived. One hundred forty-one (141, 48.6%) were men, and the median age (10th percentile, 90th percentile) was 60 (41, 80). Using logistic regression models, older age, history of hypertension, high levels of inflammatory markers, low oxygen saturation, substantial lung involvement in computed tomography (CT) scans, and gravity of the disease as indicated by the WHO 8-point ordinal scale were primary predictors of mortality at ICU. The use of remdesivir and imatinib was associated with a statistically non-significant reduction in mortality. The use of tocilizumab had almost no effect on mortality.

**Conclusion:**

The findings are consistent with and add to the currently existing international literature. The findings may be used to predict risk of mortality from COVID-19 and provide some guidance on potential treatments.

## Introduction

 As of August 4, 2022, 582 million confirmed cases and 6.4 million deaths due to the coronavirus disease 2019 (COVID-19) have been reported worldwide.^1^ The pandemic started in Wuhan, China, in December 2019 and quickly spread to many other countries. Iran was one of the first countries to report a major COVID-19 epidemic, with the epidemic starting to overwhelm the hospitals as early as March 2020. Thus far, over 7.4 million confirmed cases and 142 thousand deaths have been reported in Iran,^1^ making Iran one of the major foci of the pandemic. Analyses of excess mortality during the pandemic show that the true numbers of infections and deaths worldwide substantially exceed the reported numbers,^2^ more so in the early stages of the epidemic and in low-income countries.^3^

 Because of the gravity of the disease in a fraction of the patients, intensive care units (ICUs) were especially hit hard during the pandemic, with patient overflows during the peaks of incidence. Learning about the predictors of ICU outcomes helps with the care of the patient and treatment modalities. While there have been some previous studies,^4^ there is an overall dearth of data from ICUs in Iran.

 In this study, we collected extensive data from a sizable number of patients admitted to the ICUs of one of the tertiary referral hospitals in Tehran, Iran, to investigate the predictors of ICU mortality, as compared to live discharge from the ICU.

## Materials and Methods

###  Setting, Design, and Study Population

 The study population included COVID-19 patients who were consecutively admitted to the ICUs of the Sina hospital from May 5, 2021, to December 6, 2021. Sina hospital is a tertiary referral hospital affiliated with the Tehran University of Medical Sciences; it is the oldest hospital in Tehran and has six ICUs, two of which were dedicated to COVID-19 patients during the pandemic. The period of data collection included the peak of the epidemic of the delta (δ) variant.

 In all, 406 patient records were reviewed, of whom 290 were enrolled in the study and 116 were excluded. Patients were excluded from the study (n = 116) because they either stayed alive for less than two days, or they were admitted to the ICU with mild to moderate COVID-19 but with other major diseases that could be considered the primary cause of death. For example, patients with the following conditions at baseline were excluded: end-stage metastatic cancers (e.g., sarcoma, uterine cancer, adrenal cancer, encephalitis, brain aneurysm, myocardial infarction, heart failure, aortic aneurysm, end-stage cirrhosis, bowel obstruction, mesenteric ischemia, severe peritonitis, and necrotizing fasciitis). These exclusions allowed the study to focus more on the causes of death that were primarily due to COVID-19.

###  Data Collection and Variables 

 Demographic data, history of prior chronic diseases, laboratory data (including markers of inflammation), radiologic data, and medication data were collected. For this study, the focus was on the following variables:

- Demographic data: sex, age, body mass index (BMI);
- Past medical history: hypertension, diabetes, ischemic heart disease, COVID-19 vaccination;
- Baseline laboratory data: white blood cell (WBC) counts, percentage of neutrophils, percentage of lymphocytes, platelet counts, lactate dehydrogenase (LDH), troponin, C-reactive protein (CRP), D-dimer, O_2_ saturation;
- Baseline radiological data at the emergency room admission as well as during the ICU admission: Severity of lung involvement on the computed tomography (CT) scan (mild/moderate vs moderately severe/severe);
- Medications: remdesivir, imatinib, and tocilizumab in the ICU for the treatment or control of COVID-19;
- The World Health Organization (WHO) 8-point ordinal scale, including the following: 1: ambulatory, no activity limitation; 2: ambulatory, activity limitation; 3: hospitalized, no oxygen therapy; 4: hospitalized, oxygen mask or nasal prongs; 5: hospitalized, noninvasive mechanical ventilation (NIMV) or high-flow nasal cannula (HFNC); 6: hospitalized, intubation and invasive mechanical ventilation (IMV); 7: hospitalized, IMV + additional support such as pressors or extracardiac membranous oxygenation (ECMO); 8: death;
- Outcome: Death in the ICU (vs. alive discharge).

All data were collected from the patient charts by two medical doctors. Variables to be collected were determined in the protocol and the forms prior to the study initiation.

###  Statistical Analysis

 After data cleaning, descriptive statistics were generated, including medians (10^th^, 90^th^ percentiles) for continuous variables and frequencies and percentages for categorical variables. The association of the important predictors with the outcome of interest was evaluated using logistic regression models. For continuous variables (e.g. age, WBC count, and LDH), the associations were investigated first using continuous values and then using quartiles.

 Variables that were statistically significantly associated with the outcome were included in a multiple logistic regression model. In the multiple regression models, *P* values for trend were calculated first using continuous variables with their original value (*P*-trend_1_) and then using continuous variables as quartile ranks (*P*-trend_2_).

## Results

###  Descriptive Results

 In all, 290 patients were enrolled in this study. Of these, 187 (64.5%) died and 103 (35.5%) survived. One hundred forty-one (141, 48.6%) were men, and the median age (10^th^ percentile, 90^th^ percentile) values were 60 (41, 80). The median BMI (10^th^ percentile, 90^th^ percentile) values were 27.3 (22.9, 34.9) kg/m^2^.

 History of hypertension (115 of 278, 41.4%), diabetes mellitus (97 of 286, 33.9%), and ischemic heart disease (51 of 290, 17.6%) were reported among the study participants. History of at least one of the above conditions was reported in 163 of 290 (56.2%) of the patients. History of COVID-19 vaccination was reported in 59 of 288 (20.5%) of the patients. The WHO ordinal scale was high ( > 4) in 157 of 290, (54.1%) and low ( ≤ 4) in 133 (45.9%) of the patients at ICU admission.

 The median (10^th^ percentile - 90^th^ percentile) values for the selected baseline ICU admission laboratory findings were: WBC (9050, 4510–16300), neutrophil percentage (87.6%, 76.7%–94.0%), lymphocyte percentage (7.6%, 3.2%–16.9%), ESR (52, 13–100), platelet counts (217 000, 121 000–340 000), CRP (76.2, 17.5–123.4), LDH (966, 628–1666), troponin (23.5, 2.4–951), and D-dimer (600, 244–3776).

 CT scan results at baseline admission to ICU showed 36 (12.4%) mild to moderate and 254 (87.6%) moderately severe to severe involvement of the lungs.

 Of the 290 patients, 269 (92.8%) received remdesivir, 132 (45.5%) received imatinib, and 42 (14.5%) received tocilizumab for treatment during the ICU hospitalization.

###  Predictors of death in the ICU

 In bivariable models, age (OR = 1.41, 95% CI: 1.17–1.68, for each 10-year increase), history of hypertension (1.94, 1.15–3.25), high WHO ordinal scale (4.17, 2.50 – 6.97), O_2_ saturation (0.94, 0.91–0.97, per 1% increase ), WBC (1.11, 1.04–1.18, per 1000 increase), percentage of neutrophils (1.06, 1.02 - 1.09, per 1% increase), LDH (1.16, 1.03–1.30, per 100-unit increase), D-dimer (1.17, 1.02–1.35, per 1000-unit increase), and moderately severe/severe CT scan findings (3.84, 1.85 – 7.98) were associated with odds of death at ICU.

 By contrast, sex (OR = 1.28, 95% CI: 0.79–2.07, comparing males to females), the use of imatinib (0.78, 0.48–1.26), remdesivir (0.71, 0.27–1.88), and tocilizumab (0.99, 0.50–1.96), previous history of vaccination (0.84, 0.47–1.51), BMI (0.99, 0.95–1.04, per kg/m^2^), history of diabetes mellitus (1.51, 0.89–2.57), history of ischemic heart disease (1.77, 0.89–3.49), platelet counts (1.00, 1.00–1.00, per 1000 increase), CRP (1.04, 0.98–1.10, per 10-unit increase), and troponin (1.03, 0.98–1.07, per 10-unit increase) were not associated with a change in the odds of death at ICU.

Table 1 shows the results of the variables that were associated with odds of death, as well as sex. Medians (10^th^–90^th^ percentiles) are shown for continuous variables and numbers and percentages are shown for categorical variables. The directions are as expected. Table 1 also shows the results of unadjusted logistic regression, with continuous variables categorized into quartiles. The results with quartiles (age, WBC, neutrophil percentage, LDH, and O_2_ saturation) are similar to continuous treatment of these variables and show a dose-response relationship with death at ICU.

**Table 1:** Predictors of Mortality in Intensive Care Unit

	**Alive**	**Dead**	**Unadjusted OR (95% CI)**	**Adjusted OR (95% CI)**
Age (n = 290, year)	57 (36–75)	64 (45–82)		
Q1			1.00	1.00
Q2			1.33 (0.68–2.64)	1.37 (0.55–3.40)
Q3			1.67 (0.86–3.25)	1.34 (0.54–3.31)
Q4			2.84 (1.41–5.76)	2.71 (1.03–7.18) P-trend_1_ = 0.004 P-trend_2_ = 0.001
Gender (n = 290)				
Female	57 (38%)	92 (62%)	1.00	1.00
Male	46 (33%)	95 (67%)	1.28 (0.79–2.07)	1.91 (0.99–3.65) P-trend_1_ = 0.109 P-trend_2_ = 0.073
Hypertension (n = 278)				
No	68 (42%)	95 (58%)	1.00	1.00
Yes	31 (27%)	84 (73%)	1.94 (1.58–3.25)	2.89 (1.35–6.18) P-trend_1_ = 0.023 P-trend_2_ = 0.007
WHO ordinal scale (n = 290)				
Low ( ≤ 4)	70 (53%)	63 (47%)	1.00	1.00
High ( > 4)	33 (21%)	124 (79%)	4.17 (2.50–6.97)	4.43 (2.33–8.42) P-trend_1_ < 0.001 P-trend_2_ < 0.001
WBC (n = 281, 1000s)	7.7 (4.1–12.6)	9.7 (5.2–17.3)		
Q1			1.00	1.00
Q2			1.78 (0.91–3.50)	1.22 (0.51–2.94)
Q3			1.93 (0.97–3.82)	1.17 (0.46–2.97)
Q4			4.26 (2.01–9.03)	2.74 (0.99–2.58) P-trend_1_ = 0.039 P-trend_2_ = 0.061
Neutrophil (n = 283, %)	86 (74–92)	89 (80–95)		
Q1			1.00	1.00
Q2			1.71 (0.88–3.35)	1.63 (0.68–3.89)
Q3			2.60 (1.30–5.20)	1.88 (0.73–4.85)
Q4			3.74 (1.81–7.78)	1.74 (0.62–4.90) P-trend_1_ = 0.216 P-trend_2_ = 0.343
LDH (n = 119)	806 (515–1332)	1070 (695–1817)		
Q1			1.00	-
Q2			1.85 (0.65–5.23)	-
Q3			2.50 (0.86–7.27)	-
Q4			5.36 (1.61–17.9)	-
D-dimer (n = 126)	923 (263–3203)	1502 (336–10345)		
Q1			1.00	-
Q2			2.31 (0.84–6.34)	-
Q3			1.92 (0.70–5.26)	-
Q4			9.69 (2.73–34.4)	-
O_2_ saturation (n = 279)	85 (70–90)	80 (62–89)		
Q1			1.00	1.00
Q2			0.47 (0.21–1.05)	0.57 (0.22–1.44)
Q3			0.36 (0.16–0.80)	0.45 (0.17–1.17)
Q4			0.18 (0.08–0.39)	0.24 (0.09–0.66) P-trend_1_ = 0.004 P-trend_2_ = 0.005
CT Scan (n = 290)				
Mild/Moderate	23 (64%)	13 (36%)	1.00	1.00
Severe	80 (31%)	174 (69%)	3.85 (1.85–7.98)	2.47 (0.94 – 6.49) P-trend_1_ = 0.071 P-trend_2_ = 0.075

* For continuous variables, median (10^th^ – 90^th^ percentile) and for categorical variables numbers (percentages) have been presented. ** To calculate P-trend_1_, continuous variables were treated as such, whereas for P-trend_2_, continuous variables were categorized as quartiles.

 Data were available for 95% of the patients (n ≥ 276) for all of these variables, except for LDH (n = 119, 41%) and D-dimer (n = 126, 43%). Therefore, all variables except LDH and D-dimer were included in the multiple logistic regression. In the multiple logistic regression models, the directions of the adjusted results remained the same as those of the unadjusted results; however, some associations were attenuated. In particular, the associations were attenuated for WBC, neutrophil count, and CT scan results.* P* values for trend are shown; *P* for trend_1_ shows the results when the continuous variables were used as such, and *P* for trend_2_ when the continuous variables were categorized as quartiles. All associations remained statistically significant (*P* < 0.05) or close to statistically significant (*P* < 0.10), except for neutrophil percentage.

## Discussion

 In this study, we evaluated some predictors of mortality after admission to ICU due to COVID-19. We found age, history of hypertension, the WHO 8-point ordinal scale, WBC, low O_2_ saturation, and substantial lung involvement as determined by CT scanning to be the predictors of mortality. In addition, high WBC, a shift to the left, and other markers of high inflammation such as high serum LDH and D-dimer were also predictors of morality, although the two latter markers were not entered in the multiple logistic regression models.

 The majority of our findings are consistent with those of other studies of risk factors of admission to the hospital due to COVID-19, as well as mortality in COVID-19 patients after admission to emergency rooms, hospitals, and ICUs. However, the currently existing literature is primarily from countries other than Iran.

 In our study, older age was a statistically significant and clinically relevant predictor of mortality at ICU. Other studies have found similar findings.^5–7^ For example, in a study of over 5000 COVID-19 patients in New York, United States, age was a strong predictor of hospitalization and critical illness.^6^ Likewise, in a study of 239 patients hospitalized for COVID-19 infection in Lombardy, Italy, age was a strong predictor of deterioration.^7^ A review of the literature by Gallo Marin et al found age to be a very consistent predictor of the gravity of disease in COVID-19 patients.^8^

 Although in our study the association between sex and mortality was not statistically significant, there was a trend toward higher death among men in adjusted analyses. Some previous studies^9–11^ have also shown that the severity of disease is generally higher and prognosis is poorer in men.

 Comorbid conditions, such as history of heart failure, hypertension, and diabetes have been shown to be major predictors of mortality from COVID-19.^8,11^ In our study, a history of hypertension was associated with a statistically significant increase in COVID-19 mortality at ICU. For diabetes and ischemic heart disease, while the point estimates were above 1, statistical significance was not reached.

 High levels of serum inflammatory markers have also been associated with worse prognosis in COVID-19 patients.^8,11^ In our study, high WBC, a shift to the left, high D-dimer levels, and high LDH were all associated with an increased risk of death at ICU.

 Several medications, such as remdesivir, tocilizumab, and imatinib have been used to improve the prognosis of COVID-19 in outpatient and inpatient clinical settings.^12–15^ While some studies have shown a benefit with the use of these medications, the effects have not been consistent or strong. For example, in the first published Phase III randomized clinical trial of remdesivir, only days of hospital stay were significantly reduced in COVID-19 patients, but mortality was not statistically significantly reduced.^12^ The use of tocilizumab to reduce mortality in COVID-19 patients has been controversial, with results being inconsistent across studies.^13,14^ For example, Stone and colleagues found tocilizumab to have almost no effect on preventing intubation and death in moderately ill COVID-19 patients.^13^ In another study, Salama and colleagues showed that in hospitalized COVID-19 patients, use of tocilizumab reduced the likelihood of progression to the composite outcome of mechanical ventilation or death, but did not improve survival.^14^ De Brabander and colleagues^15^ showed imatinib to be effective in reducing mortality in a subgroup of COVID-19 patients, i.e., those with alveolar epithelial injury indicated by increased surfactant protein D levels in the context of systemic inflammation and endothelial dysfunction. In our study, use of none of the above-mentioned medications was associated with reduced mortality. However, remdesivir and imatinib showed a trend toward improvement, which may indicate that they may be useful in a subgroup of patients.

 A high degree of lung involvement, as indicated by low O_2_ saturation or CT scan findings (moderately severe/severe) was another predictor of mortality in patients admitted to the ICU. This finding is consistent with the literature from Iran^16^ and elsewhere.^8,10^

 The WHO 8-point ordinal scale is a strong predictor of death in COVID-19 patients.^12,17,18^ In our study, we did not have very low scores, as the patients were already admitted to the ICU. When categorized into two groups, this scale was highly predictive of death, even after adjustment for other variables. The results are consistent with other studies in the literature.^12,17,18^

 This study has some strengths and limitations. The strengths include a sizable number of patients; availability of data for clinical history, medications, laboratory findings, and radiologic findings; and comparisons within one hospital, which might increase internal validity. The primary limitations include the retrospective nature of data collection and lack of complete data for all patients.

 In summary, older age, history of hypertension, high levels of inflammatory markers, low oxygen saturation, substantial lung involvement, and gravity of the disease as indicated by the WHO ordinal scale were primary markers of mortality at ICU. The use of remdesivir and imatinib was associated with a statistically non-significant reduction in mortality. The use of tocilizumab had almost no effect on mortality. These results are largely consistent with the existing literature, add to and confirm previous findings, and may provide some guidance for treatment in ICUs.
